# Desflurane consumption during automated closed-circuit delivery is higher than when a conventional anesthesia machine is used with a simple vaporizer-O_2_-N_2_O fresh gas flow sequence

**DOI:** 10.1186/1471-2253-8-4

**Published:** 2008-07-17

**Authors:** Sofie De Cooman, Nathalie De Mey, Bram BC Dewulf, Rik Carette, Thierry Deloof, Maurice Sosnowski, Andre M De Wolf, Jan FA Hendrickx

**Affiliations:** 1 Department of Anesthesiology, Institut Jules Bordet, Université Libre de Bruxelles (U.L.B.), Brussels, Belgium; 2 Department of Anesthesiology, Intensive Care and Pain Therapy, Onze Lieve Vrouw Hospital, Aalst, Belgium; 3 Department of Anesthesiology, Feinberg School of Medicine, Northwestern University, Chicago, Illinois, USA

## Abstract

**Background:**

The Zeus^® ^(Dräger, Lübeck, Germany), an automated closed-circuit anesthesia machine, uses high fresh gas flows (FGF) to wash-in the circuit and the lungs, and intermittently flushes the system to remove unwanted N_2_. We hypothesized this could increase desflurane consumption to such an extent that agent consumption might become higher than with a conventional anesthesia machine (Anesthesia Delivery Unit [ADU^®^], GE, Helsinki, Finland) used with a previously derived desflurane-O_2_-N_2_O administration schedule that allows early FGF reduction.

**Methods:**

Thirty-four ASA PS I or II patients undergoing plastic, urologic, or gynecologic surgery received desflurane in O_2_/N_2_O. In the ADU group (n = 24), an initial 3 min high FGF of O_2 _and N_2_O (2 and 4 L.min^-1^, respectively) was used, followed by 0.3 L.min^-1 ^O_2 _+ 0.4 L.min^-1 ^N_2_O. The desflurane vaporizer setting (F_D_) was 6.5% for the first 15 min, and 5.5% during the next 25 min. In the Zeus group (n = 10), the Zeus^® ^was used in automated closed circuit anesthesia mode with a selected end-expired (F_A_) desflurane target of 4.6%, and O_2_/N_2_O as the carrier gases with a target inspired O_2_% of 30%. Desflurane F_A _and consumption during the first 40 min were compared using repeated measures one-way ANOVA.

**Results:**

Age and weight did not differ between the groups (P > 0.05), but patients in the Zeus group were taller (P = 0.04). In the Zeus group, the desflurane F_A _was lower during the first 3 min (P < 0.05), identical at 4 min (P > 0.05), and slightly higher after 4 min (P < 0.05). Desflurane consumption was higher in the Zeus group at all times, a difference that persisted after correcting for the small difference in F_A _between the two groups.

**Conclusion:**

Agent consumption with an automated closed-circuit anesthesia machine is higher than with a conventional anesthesia machine when the latter is used with a specific vaporizer-FGF sequence. Agent consumption during automated delivery might be further reduced by optimizing the algorithm(s) that manages the initial FGF or by tolerating some N_2 _in the circuit to minimize the need for intermittent flushing.

## Background

Low flow techniques reduce anesthetic agent consumption but are often perceived as cumbersome. First, frequent vaporizer (F_D_) or fresh gas flow (FGF) adjustments are thought to be needed, especially at the beginning, when initial wash-in and high uptake by the patient rapidly alter the concentrations of anesthetic gases in the circle system. Second, when FGF is lowered below minute ventilation, a discrepancy develops between the delivered and the inspired concentrations of gases and vapors, which has been considered as "lack of control". Finally, the older literature has been preoccupied by the use of rather complex uptake models to administer these agents with closed-circuit anesthesia (CCA) techniques. The administration schedules in the appendix of the monograph by Lowe and Ernst are daunting to the clinician [1]. The Zeus^® ^anesthesia machine (Dräger, Lübeck, Germany) black-boxes these issues for the clinician by administering inhaled agents by automated closed-loop end-expired feedback [2]. However, the Zeus^® ^also *has *to use a high FGF, initially to wash-in the circuit and the lungs, and later to intermittently flush the circuit to remove unwanted N_2_. Because this use of high FGF periods increases desflurane consumption above true CCA conditions, we hypothesized that desflurane consumption with a conventional anesthesia machine (ADU^® ^or Anesthesia Delivery Unit, GE, Helsinki, Finland) might be lower than with the Zeus^® ^if used with a previously derived desflurane-O_2_-N_2_O F_D_-FGF schedule that allows very early FGF reduction [3,4]. The desflurane concentrations resulting from the use of that particular administration schedule with the ADU anesthesia machine have been presented [4], and in this manuscript we present only those data pertinent to the current study.

## Methods

After obtaining IRB approval and informed consent, 36 ASA PS I or II patients undergoing plastic, urologic, or gynecologic surgery were enrolled. All patients received oral alprazolam 1 h before the scheduled start of surgery. After preoxygenation (8 L.min^-1 ^O_2 _FGF for 3 min), propofol (3 mg.kg^-1^), rocuronium (0.7 mg.kg^-1^), and sufentanil (0.1 μg.kg^-1^) were administered intravenously. After intubation of the trachea, ventilation was mechanically controlled. Initial tidal volume and respiratory rate was 500 mL and 10 breaths per minute, respectively; these settings were later adjusted at the discretion of the attending anesthesiologist. Anesthesia was maintained with desflurane in O_2_-N_2_O, and additional sufentanil was allowed to be given. Patients were assigned to one of two groups, depending on the anesthesia machine and low flow technique that was used.

In the ADU group (n = 26), an ADU anesthesia machine (Anesthesia Delivery Unit, GE, Helsinki, Finland) was used. Desflurane and O_2_-N_2_O were administered with a particular two-step vaporizer-FGF sequence, details of which have been previously described [4]. Briefly, an initial 3 min high FGF of O_2 _and N_2_O (2 and 4 L.min^-1^, respectively) was followed by 0.3 L.min^-1 ^O_2 _+ 0.4 L.min^-1 ^N_2_O. In patients weighing more than 95 kg, a 4 min high FGF period was used. Desflurane F_D _was 6.5% for the first 15 min, and 5.5% during the next 25 min. In- and expiratory gases were analyzed by a multigas analyzer (Datex-Ohmeda Compact Airway Module M-CAiOV, Datex-Ohmeda, Helsinki, Finland) and downloaded in a spreadsheet every minute. Gases sampled by the multigas analyzer (200 mL.min^-1^) were not redirected to the anesthesia circuit to avoid N_2 _accumulation [5]. In the Zeus group (n = 10), the automated CCA mode was selected with a desflurane F_A _target of 4.6% and O_2_-N_2_O as the carrier gas with an F_I_O_2 _target of 30%; 4.5% could not be selected because the desflurane target can only be increased with 0.2% increments. Concentrations of the in- and expiratory gases and agent use were downloaded via the proprietary software in a spreadsheet every minute.

Desflurane consumption with the Zeus^® ^machine was retrieved form the amount of agent the injector has injected into the anesthesia circle system, information that was downloaded to a laptop computer in an Excel file every 10 seconds. With the ADU^®^, desflurane consumption was first calculated as mL vapor from FGF and vaporizer output (not dialed concentrations); this amount of vapor is easily converted to mL liquid desflurane (1 mL liquid desflurane equals 209.3 mL vapor at 20°C). The same ADU anesthesia machine was used throughout the study. Vaporizer output was intermittently measured at the common gas outlet, and could be related to the dialed concentration as [desflurane vaporizer output (%) = 0.10 + 0.97* vapor setting] – see reference 4 for details.

All results are presented as mean ± standard deviation. Desflurane consumption (retrieved directly from both machines) and desflurane F_A_during the first 40 min were compared using repeated measures one-way ANOVA, with P < 0.05 indicating statistical significance.

## Results

Age, height, weight, and Body Mass Index (BMI) were 54 ± 12 years, 167 ± 8 cm, 73 ± 12 kg, and 24 ± 6 kg/m^2 ^in the ADU and 51 ± 16 years, 174 ± 9 cm, 73 ± 17 kg, and 24 ± 4 kg/m^2 ^the Zeus group, respectively. Age, weight, and BMI did not differ between the groups (P > 0.05), but patients in the Zeus group were taller (P = 0.04).

**Figure 1:**
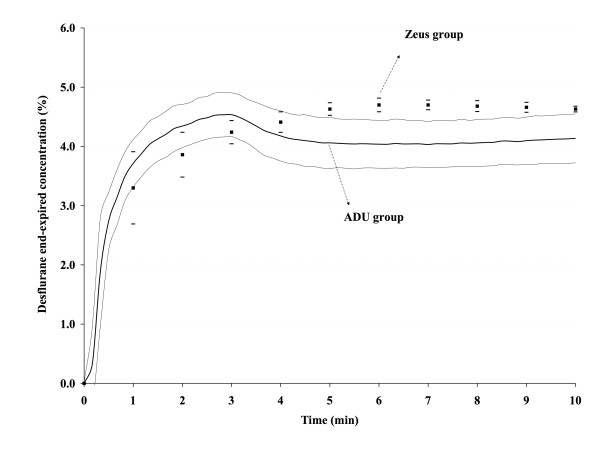
**End-expired desflurane concentrations (F_A_, %) with the vaporizer-fresh gas flow sequence (ADU group) or with automated closed-circuit anesthesia (Zeus^® ^group).** Results are presented as mean ± standard deviation.

**Figure 2:**
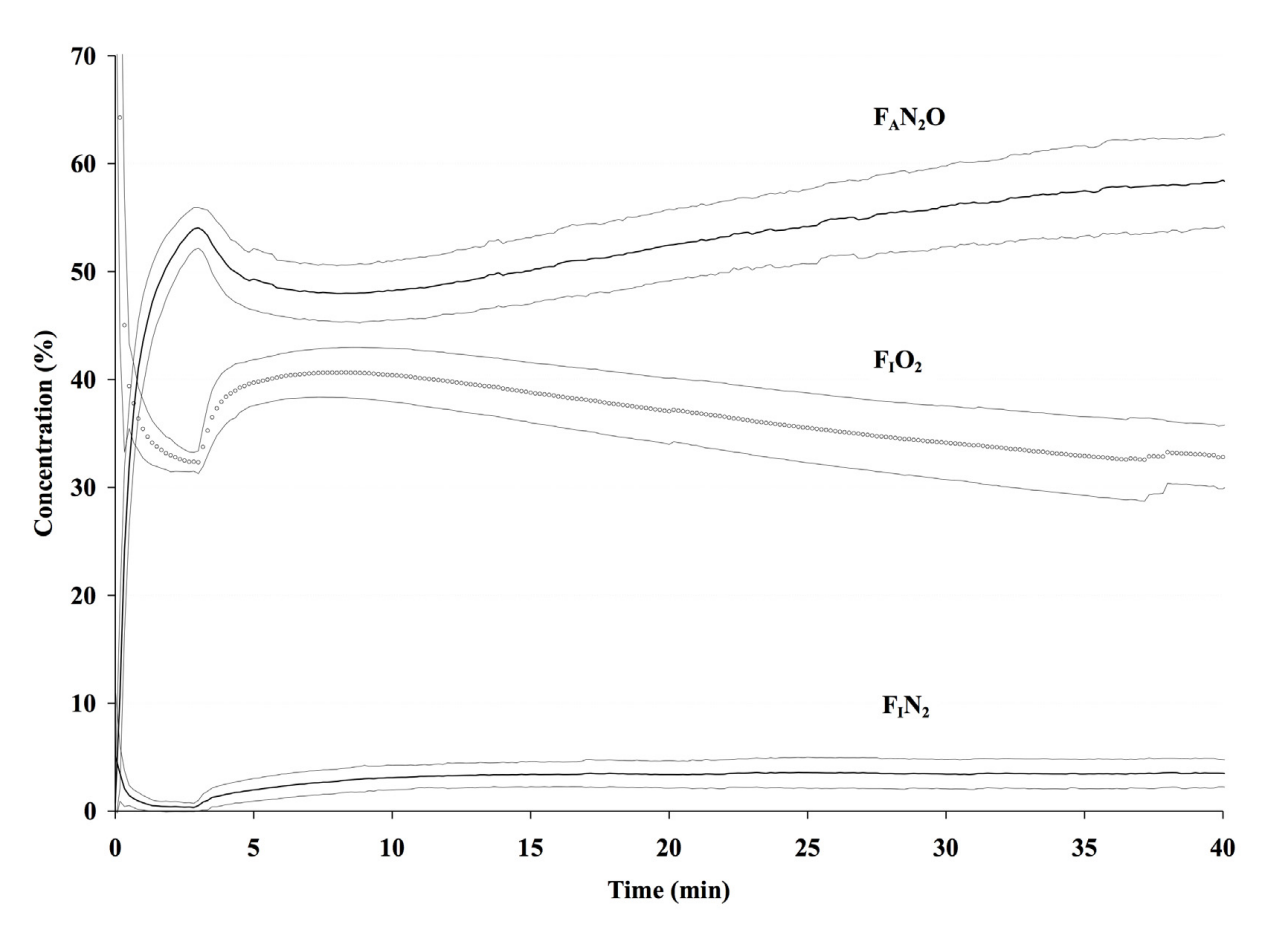
**Inspired O_2 _(F_I_O_2_), end-expired N_2_O (F_A_N_2_O), and inspired N_2 _(F_I_N_2_) concentrations with a conventional anesthesia machine (ADU^®^).** Results are presented as mean ± standard deviation.

In the ADU group, F_A_des was 3.71 ± 0.40% after 1 min, reached 4.53 ± 0.38% after 2 min and 40 seconds, and fell to a nadir of 4.03 ± 0.39% 4 min after lowering the FGF (figure 1); after 15 min, F_A_des had gradually increased to 4.40 ± 0.44% and remained almost constant during the ensuing 30 min (4.50 ± 0.36% after 45 min). The average desflurane F_A _of all patients combined between 5 and 40 min was 4.41% (standard deviation 0.43), slightly lower than the 4.6% target in the Zeus group. The course of F_A_N_2_O and F_A_N_2 _in the ADU group are presented in figure 2.

**Figure 3:**
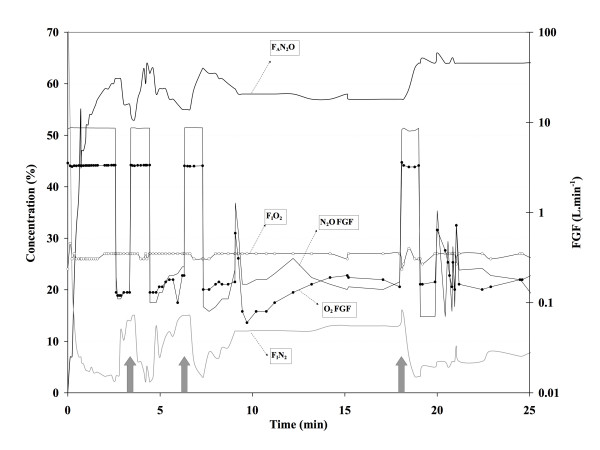
**Example of fresh gas flow management by the Zeus^® ^and resulting inspired O_2 _(F_I_O_2_), end-expired N_2_O (F_A_N_2_O), and N_2 _(F_I_N_2_) concentrations.** Note how during automated administration the FGF of O_2 _and N_2_O (right Y-axis, logarithmic scale) is increased whenever F_I_N_2 _increases above a threshold value of approximately 15% (grey arrows).

In the Zeus group, desflurane F_A _(figure 1) was lower than in the ADU group during the first 3 min (P < 0.05), identical at 4 min (P > 0.05), and slightly higher after 4 min (P < 0.05). The F_A _target was maintained at 4.6% after 4 min in all patients in the Zeus group. In the Zeus group, the course of F_A_N_2_O is less consistent between patients than in the ADU group (compare figures 2 and 3) because high and low FGF alternate, steered by the proprietary algorithms of the Zeus^® ^to combine the goals of achieving the desired target concentrations of the anesthetic gases and minimizing agent and gas consumption. A typical example of fresh gas flow management by the Zeus^® ^and resulting in-expired O_2_(F_I_O_2_), end-expired N_2_O (F_A_N_2_O), and inspired N_2 _(F_I_N_2_) concentrations are presented in figure 3.

Desflurane consumption was higher in the Zeus than in the ADU group at all times (Table 1); this difference persisted after correcting for the small difference in F_A _between the two groups.

**Table 1:** Desflurane consumption (cumulative dose, mL liquid) was higher at all times (P < 0.05) during automated closed-circuit anesthesia administration (Zeus^®^) than with a conventional machine used with the predefined low flow sequence (ADU^®^).

Time (min)	ADU group	Zeus group
0	0.0 ± 0.0	0.0 ± 0.0
5	6.0 ± 0.0	11.7 ± 4.0
10	7.1 ± 0.0	12.9 ± 4.1
15	8.2 ± 0.0	13.9 ± 4.2
20	9.1 ± 0.2	14.8 ± 4.5
25	10.0 ± 0.3	15.4 ± 4.3
30	11.0 ± 0.3	15.9 ± 4.5
35	11.9 ± 0.3	16.6 ± 4.6
40	12.8 ± 0.4	17.2 ± 4.8

## Discussion

The use of a specific desflurane-O_2_-N_2_O F_D_-FGF sequence with a conventional anesthesia machine provides comparable end-expired desflurane concentrations and reduces desflurane consumption below that with an automated CCA machine, or alternatively, desflurane consumption during automated closed-circuit delivery is higher than with a conventional anesthesia machine if the latter is used with a particular vaporizer-O_2_-N_2_O fresh gas flow sequence.

The performance of the Zeus^® ^has been described in vitro, and compared with the Primus^® ^(Dräger, Lübeck, Germany) [2]. The target desflurane F_A _was attained slightly later with the Zeus^® ^because part of the fresh gas goes directly to the patient with the Primus^®^, while fresh gas is extensively mixed in the circle system before reaching the patient in the Zeus^® ^[2]. The ADU^® ^design (with the FGF inflow located just distal to the inspiratory valve) also explains the slightly faster rise of F_A _desflurane in the ADU group. After wash-in, F_A _desflurane is maintained within a slightly narrower range with the Zeus^® ^than with the 0.7 L.min^-1 ^FGF with the ADU^® ^(figure 1). More details regarding the performance characteristics of the F_D_-FGF sequence can be found elsewhere [4].

Even though it is well known that the initial high FGF period is a major determinant of vapor and gas consumption, many anesthesiologists continue to use high FGF initially because low flow techniques have been perceived as particularly cumbersome to use at the beginning of an anesthetic. Because the initial wash-in and high uptake by the patient rapidly alter the concentrations of anesthetic gases in the circle system, the need for frequent vaporizer and rotameters adjustments required when using low FGF has been claimed to be too distracting at a time when the anesthesiologist is preoccupied by other tasks. Like Lerou [6], we therefore argue that a simple, clinically easy to apply F_D_-FGF schedule as used in our ADU group could encourage anesthesiologists to more frequently use lower FGF early on during the anesthetic. With a conventional machine with ascending visible bellows (e.g. the ADU^®^), the acceptance of a small temporary bellows deficit allows for early FGF reduction (after 3 min), and the small amount of FGF in excess of patient uptake and gas sampling during maintenance slowly washes out N_2 _yet contributes little to overall desflurane consumption. The somewhat unexpected finding that agent consumption is higher with the Zeus^® ^than with the ADU^® ^is the result of two factors: the use of a very high initial FGF (> 11 L/min) and the intermittent flushing of the gas reservoir when N_2 _concentration reaches a certain threshold. The effect of the first factor is readily demonstrated by calculating the effect on desflurane consumption of shortening the high FGF period by one min. With the described F_D_-FGF sequence with the ADU^®^, desflurane consumption is 12.8 mL after 40 min; when the duration of high FGF is extended by one min, total consumption increases to 14.4 mL. If the high FGF would be extended by another 2 minutes, desflurane consumption would become similar to that with the Zeus^®^. The second important factor that explains the difference in agent consumption is the difference in the manner the two techniques handle N_2_. With the conventional anesthesia machine, gases sampled by the agent analyzer (200 mL.min^-1^) are not redirected to the anesthesia circuit (avoiding air entrainment by the gas analyzer into the circuit at a rate of 32 m L.min^-1 ^[5]), and the 0.7 L.min^-1 ^maintenance FGF used with the F_D_-FGF sequence therefore ensures that N_2 _concentration in the circuit remains very low and even continues to decrease despite a relatively short high FGF period (figure 2). The Zeus^® ^takes a different approach. As soon as possible and whenever possible, it will try to convert to CCA conditions. Closing the circuit will inevitable lead to a subsequent increase in N_2 _in the anesthesia circuit because vessel rich tissues are still releasing significant amounts of N_2_. Whenever the F_I_N_2 _increases above a certain threshold (e.g. F_I_N_2 _of 15%), FGF is increased to flush the circuit (figure 3). Our data indicate that serial flushing to reduce N_2 _increases agent consumption well above the amount taken up by the patient, to the extent that desflurane consumption with our current F_D_-FGF sequence used with a conventional anesthesia machine and a FGF in between minimal flow (0.5 L.min^-1^) and low flow (1.0 L.min^-1^) becomes lower than that with an automated closed-circuit anesthesia machine, that – according to the definition of closed circuit anesthesia – should administer just that amount of agent that is taken up by the patient and lost via circuit leaks. This suggests that administration regimens similar to ours could help steer more conventional anesthesia machines to lower agent consumption similar to or below that of automated closed-loop feedback anesthesia machines, or could be used to optimize administration algorithms of automated closed-loop feedback anesthesia machines themselves. Agent consumption by the Zeus^® ^could be further reduced by optimizing the algorithm that manages the initial FGF (e.g. by allowing a more gradual rise towards the desired anesthetic gas concentrations) or by tolerating some N_2 _in the circuit to minimize the need for high FGF used during intermittent flushing. Our findings also suggest that the actual FGFs used by a particular technique or device should always be explicitly mentioned to allow proper interpretation of terms like "closed-circuit".

## Conclusion

Anesthetic agent consumption with an automated closed-circuit anesthesia machine is higher than with a conventional anesthesia machine *if *the latter is used with a specific vaporizer-FGF sequence. More specifically, one desflurane vaporizer and one O_2_/N_2_O FGF change with a conventional anesthesia machine with ascending visible bellows suffices to maintain satisfactory anesthetic gas concentrations within a clinically sufficiently narrow range during the first 20–30 min in most patients. The higher consumption with the closed-circuit anesthesia machine is caused by the use of high FGF for initial circuit and lung wash-in and by intermittent flushing of the circuit to reduce N_2 _accumulation. We therefore suggest that agent consumption during automated delivery can be further reduced by optimizing the algorithm(s) that manages the initial FGF (e.g. by allowing a more gradual rise towards the desired anesthetic gas concentrations) or by tolerating some N_2 _in the circuit to minimize the need for intermittent flushing.

## Competing interests

The authors declare that they have no competing interests.

## Authors' contributions

Conception and design: JHX, ADW. Acquisition of data, or analysis and interpretation of data: SDC, NDM, BBCD, JHX. Drafting the manuscript or revising it critically: All authors. Given final approval of the final submitted version: All authors.

## Pre-publication history

The pre-publication history for this paper can be accessed here:


